# Characterization of the Biological Fingerprint and Identification of Associated Parameters in Stress Fractures by FTIR Spectroscopy

**DOI:** 10.1155/2019/1241452

**Published:** 2019-09-22

**Authors:** Monica Maribel Mata-Miranda, Melissa Guerrero-Ruiz, Juan Ramon Gonzalez-Fuentes, Carlos Martin Hernandez-Toscano, Jesus Rafael Garcia-Andino, Miguel Sanchez-Brito, Gustavo Jesus Vazquez-Zapien

**Affiliations:** ^1^Escuela Militar de Medicina, Centro Militar de Ciencias de la Salud, Secretaría de la Defensa Nacional, Ciudad de México 11200, Mexico; ^2^Hospital Central Militar, Secretaría de la Defensa Nacional, Ciudad de México 11200, Mexico; ^3^Doctorado en Ciencias de la Ingeniería, Instituto Tecnológico de Aguascalientes, Aguascalientes 20256, Mexico

## Abstract

**Introduction:**

The stress fractures (SFs) are a common condition in athletes and military recruits, characterized by partial fracture caused by repetitive applications of stresses that are lower than the stress required to fracture the bone in a single loading. Fourier transform infrared (FTIR) spectroscopy gives information about the bone composition and also can determine the amount of a molecule. For this reason, the FTIR spectroscopy may be used as a tool for diagnosis of certain bone diseases related to the bone strength. In this research, we established the contributions of mineral and collagen properties to SF risk through FTIR spectroscopy, analyzing the biochemical profile differences between the healthy bone and the bone with an SF.

**Materials and Methods:**

Previous written informed consent was obtained, and samples of the hip with an SF (*n* = 11) and healthy bone from the femur with traumatic fracture (*n* = 5) were obtained and analyzed employing FTIR spectroscopy and its biochemical mapping function. Then, using FTIR spectra and the map, the collagen content and ratios corresponding to matrix maturity, mineralization, carbonate substitution, acid phosphate substitution, and crystallinity were calculated. Moreover, a histopathological analysis through Masson's staining was conducted.

**Results:**

The biochemical analysis showed that the bone with an SF presented a bone immaturity characterized by a higher content of collagen, lower matrix maturity, mineralization, carbonate and acid phosphate substitutions, and greater crystallinity compared to the healthy bone, being checked by the ratio analysis and biochemical mapping. Besides, Masson's stain showed a higher collagen content in the bone with an SF.

**Conclusions:**

The bone with an SF presented alterations in its biochemical composition, showing bone immaturity, which broadens the panorama of the condition to investigate future treatments or prophylactic techniques.

## 1. Introduction

The stress fractures (SFs) are a common condition in athletes and military recruits, characterized by partial fracture caused by repetitive applications of stresses that are lower than the stress required to fracture the bone in a single loading [1, 2]. These fractures were firstly described in Prussian soldiers by Breithaupt in 1855, the reason by which these were named “march fractures.” After that, in 1958, Devas made the first report of the SF in athletes [3].

Although the precise physiopathology of the SF is unknown, it has been reported that, after repetitive bouts of mechanical loading, a bone strained is provoked, and as with other structural materials, repetitive strain in the bone is associated with the generation of damage. Nevertheless, the provoked damage is not so significant once the bone is capable of self-repairing through targeted remodeling. However, imbalances between damage generation and its removal are susceptible, bringing out the accumulation of damage, resulting in an elastic deformation that progresses to plastic deformity until it finally results in microfracture, and if this condition is not treated, it will be evolved into a bone fracture [1, 3].

Numerous factors have been associated with the risk of SF development. These factors have been grouped into two categories, extrinsic and intrinsic. Extrinsic risk factors include the type of activity or sport, equipment used, the type of ground, and nutritional habits, such as alcohol consumption (more than 10 alcoholic drinks per week), and low levels of 25-hydroxyvitamin D. On the contrary, the intrinsic risk factors include skeletal, muscle, joint, and biomechanical factors, as well as physical fitness, age, and gender, once some studies have reported that women and young people have a higher risk of the SF [1, 3–5]. Furthermore, some studies have declared that some other factors such as rigid pes cavus, discrepancy of the lower limbs, short tibia, genu valgum, increased *Q* angle, body mass index lower than 21 kg/m^2^, and short stature should also be taken into consideration into the risk factors for the SF, which make it multifactorial and difficult to control [3].

Bone strength is bone resistance to fracture. Currently, it is difficult to quantify what makes up the strength of the bone, but it is related to bone mass (amount of the bone) and bone structure (quality of the bone); the mass refers to the bone mineral density (BMD), and the bone structure denotes the bone structural and material properties [1, 6]. The bone structural properties include geometry and microarchitecture, whereas the material properties involve the mineral and collagen components [7].

In this sense, the bone capability to resist repetitive low-magnitude loads is dependent on bone strength [1]. The maximum bone size and strength are called peak bone mass. The bone reaches its maximum strength at the age of 25 to 26 years [5]. Nevertheless, few studies investigate the bone components to elucidate the biomolecular content importance in the SF.

About this, there are several techniques that allow the determination of the biochemical composition of different tissues. However, these techniques require special treatments and do not allow analysis of fresh tissue, the reason by which some other techniques have emerged, such as the Fourier transform infrared (FTIR) spectroscopy.

FTIR spectroscopy provides molecular structure information of organic and inorganic materials. In the FTIR spectroscopy analysis, absorption of infrared (IR) radiation occurs when a photon is transferred to a molecule and excites it to a higher energy state, resulting in the vibrations of molecular bonds generating a variability of wavenumbers or frequencies [8]. Importantly, biological materials like proteins, carbohydrates, lipids, and nucleic acids have a unique structure, so it is possible to obtain spectral fingerprints corresponding to their functional groups. This technique has been proven to be a fundamental and valuable technique in biology and medicine because of its high sensitivity to detect changes in the functional groups mentioned above [9].

The coupling of FTIR spectrometers with visible microscopes has led to the successful use of this technique to perform imaging analysis, in which biochemical information and spatial information of biological samples, such as tissues and cells, are combined [10].

Moreover, the ability of FTIR spectroscopy to characterize the biomolecular components such as minerals or collagen in the human bone has already been investigated; this novel technique gives information about the bone composition and also can determine the amount of a molecule. For this reason, the FTIR spectroscopy may be used as a tool for diagnosing certain bone diseases related to the bone strength.

In this research, we established the contributions of mineral and collagen properties to SF risk through FTIR spectroscopy, analyzing the biochemical profile differences between the healthy bone and the bone with an SF, once the FTIR spectroscopy has not been used to analyze the biochemical composition of the bones with SFs. We hypothesize that the FTIR spectroscopy can identify differences in tissue composition between the healthy bone and the bone from patients diagnosed with an SF.

## 2. Materials and Methods

### 2.1. Study Population

The current study was conducted at the “Hospital Central Militar” of the National Defense Ministry, Mexico, in the period from November 2017 to November 2018. Two study groups were considered, the control (healthy bone (HB)) group and the SF group. For this purpose, all the patients that attended in the period previously mentioned with a diagnosis of the SF of the hip and accepted to participate in the study integrated to form the SF group, and five patients with a diagnosis of femur traumatic fracture that would be subjected to a surgical procedure integrated to form the HB group.

Patients aged between 18 and 40 years were included in both groups. Nevertheless, patients with an infectious pathology or with other fractures were excluded from the study.

Written informed consent for the obtention of 1 cm^3^ of a bone sample from the metaphysis of the femur and participation in this study was obtained. The Institutional Research Ethics Committee approved the protocol and the informed consent forms. All experiments were examined and approved by the appropriate ethics committee and were performed following the ethical standards laid down in the 1964 Declaration of Helsinki.

### 2.2. Sample Collection

For the development of this research, the patients considered for this study were subjected to the surgical procedure from 4 to 7 days after fracture. For the sample collection, after surgical cleansing and during the surgical procedure, one bone sample of 1 cm^3^ of unaffected tissue was obtained from the metaphysis of the proximal femur, making an incision of 3 cm in the anatomic region of the major trochanter; the sample was obtained using a drill bit of 16 mm with a flexible cannulated proximal femoral nailing system (DePuy Synthes, Johnson & Johnson), highlighting that all the surgical procedures were performed by the same surgeon. Once the sample was obtained, it was kept in cold storage for its immediate analysis.

### 2.3. Fourier Transform Infrared Spectroscopy

FTIR spectral analysis was conducted in the spectral range between 4000 and 400 cm^−1^ (midinfrared), using an FTIR spectrometer (6600, Jasco) in the attenuated total reflection (ATR) sampling mode. The instrument has a fixed spectral resolution of 4 cm^−1^. The sample was deposited onto the surface of the ATR crystal and dried at room temperature for about 15 minutes to eliminate excess water. The infrared radiation propagated along the crystal to obtain the corresponding spectra which were the average of 120 data acquisitions. Three replicates of each sample were developed.

### 2.4. Bone Composition by Spectral Analysis

Spectral analysis was performed in the fingerprint region (1800–500 cm^−1^) using the Jasco Spectra Manager software (version 2.13C, Jasco). FTIR absorbance spectra were normalized using a standard normal variate (SNV) normalization employing the Unscrambler X software (version 10.3, Camo). All the spectra were averaged according to the group that they belonged (HB or SF group).

The main bone components and its material properties, such as the collagen content, matrix maturity, bone mineralization, carbonate substitution, acid phosphate substitution, and crystallinity, were calculated.

Regarding the collagen content, as previously mentioned, the the main organic part of the bone matrix is composed of type I collagen, which was assessed calculating the peak area of amide I (A1700–1580 cm^−1^).

The ratio of nonreducible to reducible collagen or mature-to-immature collagen cross-links in the bone was calculated to analyze the matrix maturity, using the second derivative spectra:

(1) $$\begin{array}{c} \text{matrix maturity}=\frac{1660\;{\text{cm}}^{-1}}{1690\;{\text{cm}}^{-1}}. \end{array}$$

The peak at 1660 cm^−1^ corresponds to the 3_10_ helix, pyridinoline collagen cross-link (mature), and the peak at 1690 cm^−1^ is related to the *β* turn, divalent collagen cross-link (immature) [7, 11].

In the same way, to analyze the bone mineralization through FTIR spectroscopy, the mineral-to-matrix ratio was calculated as follows:

(2) $$\begin{array}{c} \text{mineralization}=\frac{\text{A}1200\text{–}900\;{\text{cm}}^{-1}}{\text{A}1648\;{\text{cm}}^{-1}}, \end{array}$$

where the range A1200–900 cm^−1^ represents the amount of phosphate in the bone (mineral) and A1648 cm^−1^ the amount of amide I, mainly collagen type I (matrix) [7, 12].

The carbonate substitution ratio was also estimated using the carbonate-to-phosphate ratio (CO_3_^2−^ to PO_4_^3−^ ratio):

(3) $$\begin{array}{c} \text{carbonate substitution}=\frac{\text{A}890\text{–}850\;{\text{cm}}^{-1}}{\text{A}1200\text{–}900\;{\text{cm}}^{-1}}, \end{array}$$

where the peak area at 890–850 cm^−1^ is associated with carbonate. This ratio reflects the extent of carbonate substitution into phosphate and hydroxyl positions in the hydroxyapatite lattice [12, 13].

In the same way, the acid phosphate substitution (APS) ratio was calculated as a measure of newly precipitated crystals employing the second derivative ratio of acid phosphate content to apatite:

(4) $$\begin{array}{c} \text{APS}=\frac{\text{A}1127\;{\text{cm}}^{-1}}{\text{A}1096\;{\text{cm}}^{-1}}. \end{array}$$

The peak at 1127 cm^−1^ is attributable to the presence of acid phosphate-containing species, and the peak at 1096 cm^−1^ is related to apatite [14, 15].

Finally, to estimate the mineral crystal size and perfection, the crystallinity index (CI) was calculated as the ratio of highly crystalline apatite to poorly crystalline apatite employing the second derivative spectra [16]:

(5) $$\begin{array}{c} \text{CI}=\frac{\text{A}1030\;{\text{cm}}^{-1}}{\text{A}1020\;{\text{cm}}^{-1}}. \end{array}$$

The peak at 1030 cm^−1^ is related to the highly crystalline apatite, and the peak at 1020 cm^−1^ corresponds to poorly crystalline apatite [7, 17].

As previously mentioned, for the calculation of matrix maturity, APS, and CI, the second derivative was calculated to find the exact peak location of each component, and each ratio was defined from the areas mentioned above, making a peak fitting to the spectra in these regions. The second derivative spectra were calculated employing the Savitzky–Golay algorithm with fifteen-point windows and the second polynomial order using Unscrambler X.

### 2.5. FTIR Spectroscopic Imaging

For this analysis, bone samples were embedded in Tissue-Tek (4583, Sakura) and frozen; after that, cryosections of 10 *μ*m were obtained and mounted on a gold-coated microscope slide with a gold layer thickness of 100 nm (643246-5EA, Sigma-Aldrich). Three sections from each patient were examined.

Micro-FTIR images (FTIRIs) were collected on the FTIR microscope (IRT-5200, Jasco) fitted with a liquid nitrogen-cooled MCT (mercury, cadmium, and tellurium) detector, coupled to an FTIR spectrometer. A 16x Cassegrain objective was used. The microscope optic permitted image amplification at a ratio of 1 : 2. The absorbance spectra were acquired in the reflectance mode at a spectral resolution of 4 cm^−1^ with 120 scans coadded, and 5 × 5 points were analyzed. Biochemical images were obtained by automated mapping of multiple points (IQ mapping) of the FTIR microscope.

The displays of the microscopic view of the sample and spectrum color-coded diagram were fitted as follows: *X* (160.054–163.946) to *Y* (141.644–162.356) and *X* (160.054–163.946) to *Y* (125.644–162.356) for HB and SF samples, respectively; the color scale bars were adjusted according to the intensity that each component showed, employing the same color scale bar for each component and taking care that the colors were visible and comparable between the study groups. Bilinear interpolation was used for this analysis.

The FTIRIs employing the Spectra Manager software analyzed the same areas and ratios calculated by FTIR spectroscopy. The collagen content, using the amide I peak area (A1700–1580 cm^−1^), matrix maturity (1660/1690), mineralization (A1200–900/A1648), carbonate substitution (A890–850/A1200–900), APS (A1127 cm^−1^/A1096 cm^−1^), and CI (A1030/A1020) were visualized. Before the calculation of each component, every area used for this arithmetic operation was normalized.

### 2.6. Masson's Trichrome Stain

A Masson's trichrome stain was developed to evidence the collagen fiber distributions. For this purpose, without receiving any treatment like demineralization, the samples were fixed embedded in paraffin, and tissue sections of 4 *μ*m were obtained. After that, tissue sections were deparaffinized and rehydrated. Afterward, Masson's trichrome staining was performed according to standard methods. Stained sections were analyzed using a light microscope (Eclipse Ti-U, Nikon) and the Image-Pro Premier software (version 9.1, Media Cybernetics).

### 2.7. Statistical Analysis

All data were performed in triplicate, and all experiments were repeated at least three times. Data were presented as mean ± SD and analyzed using the Mann–Whitney *U* test to determine any significant differences. *p* values less than 0.05 were considered statistically significant.

## 3. Results

### 3.1. Description of the Study Population

The main data of the sample donors are summarized in Table 1. As shown, 11 bone samples from the femur metaphysis of patients with the diagnosis of the SF were obtained, highlighting that eight samples belonged to female patients and three to male patients; moreover, ten patients were less than 30 years old, and ten samples came from military recruits.

### 3.2. FTIR Spectroscopy Analysis

**Figure 1:**
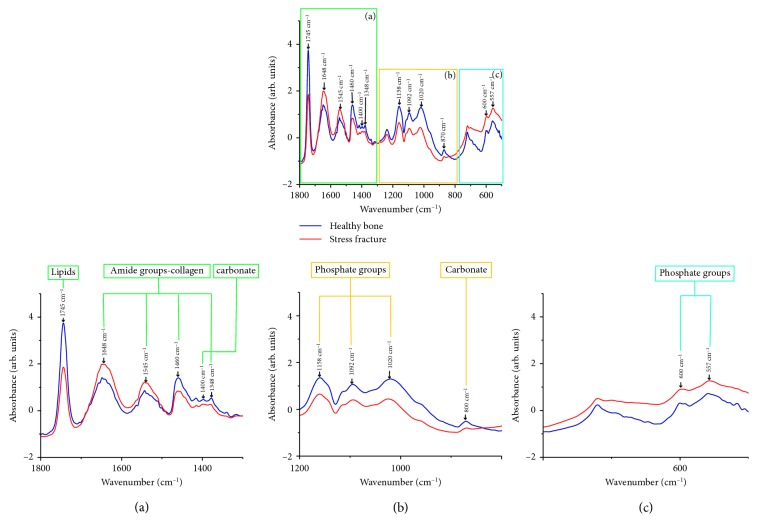
FTIR spectra of the healthy bone (HB) and bone with a stress fracture (SF) measured in the fingerprint region (1800–500 cm^−1^). Several absorption bands of lipids, proteins (collagen type I), and minerals (phosphates and carbonates) were observed. Each spectrum corresponds to the average of five samples of the HB and 11 samples of the bone with an SF, which was measured three times.

The averages of the raw and normalized FTIR spectra of the HB and SF groups are shown in Figure 1. In the biological fingerprint region (1800–500 cm^−1^), different representative bands associated with bone biomolecules are evidenced such as lipids, proteins (collagen type I), and minerals (phosphates and carbonates).

Firstly, in the region from 1800 to 1400 cm^−1^, lipids, amide groups related to collagen, and carbonates were evidenced (Figure 1(a)). At 1745 cm^−1^, the absorption bands related to the extension vibrations of the C=O ester group of lipids showed a higher absorbance in the HB group than in the SF group. Then, the peaks at 1648 and 1545 cm^−1^, which correspond to the functional groups of amide I (C=O extension), specifically to collagen type I, and amide II (CN extension + NH flexion), respectively, exhibited a higher absorbance in the SF group. Nevertheless, the bands at 1460 and 1348 cm^−1^ associated with collagen as well as the peak at 1400 cm^−1^ related to carbonate showed a higher absorbance in the HB group.

On the contrary, the range from 1200 to 800 cm^−1^ corresponding to the phosphate groups and carbonate was observed (Figure 1(b)), highlighting that all these peaks exhibited a higher absorbance in the HB group. Finally, in the region from 650 to 500 cm^−1^ also, bands related to phosphate groups were shown at 600 and 557 cm^−1^ (Figure 1(c)), which exhibited a higher absorbance in the SF group.

**Figure 2:**
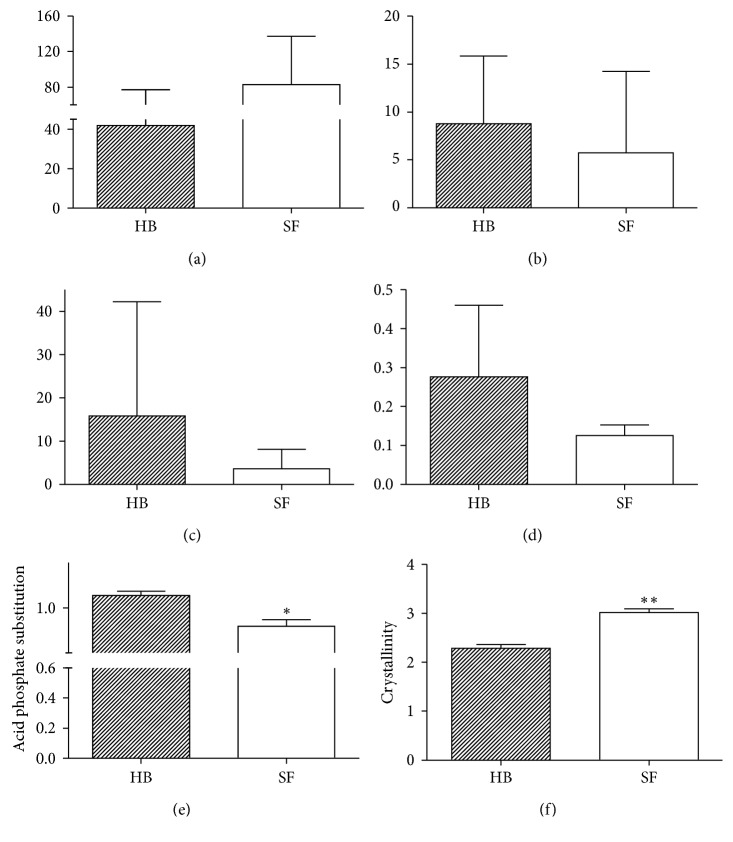
Summary of measured FTIR bone components for the (a) collagen content, (b) matrix maturity, (c) mineralization, (d) carbonate substitution, (e) acid phosphate substitution, and (f) crystallinity. ^*∗*^*p* < 0.05 and ^*∗∗*^*p* < 0.005, relative to the healthy bone (HB). SF: stress fracture.

Regarding the collagen content, a greater collagen type I content (A1700 to 1580 cm^−1^) was exhibited in the SF group than in the HB group (*p*=0.11; Figure 2(a)). On the contrary, the matrix maturity (mature-to-immature collagen cross-link ratio) was lower in the SF group (*p*=0.42; Figure 2(b)). In the same way, the mineralization (mineral-to-matrix ratio) and the carbonate substitution (carbonate-to-phosphate ratio) were lower in the SF group (*p*=0.25 and *p*=1.00; Figures 2(c) and 2(d), respectively). However, the APS was significantly lower in the SF group (*p*=0.014; Figure 2(e)), whereas the CI (highly crystalline apatite-to-poorly crystalline apatite ratio) was significantly higher in the SF group (*p* < 0.006; Figure 2(f)).

### 3.3. IQ Mapping Analysis

**Figure 3:**
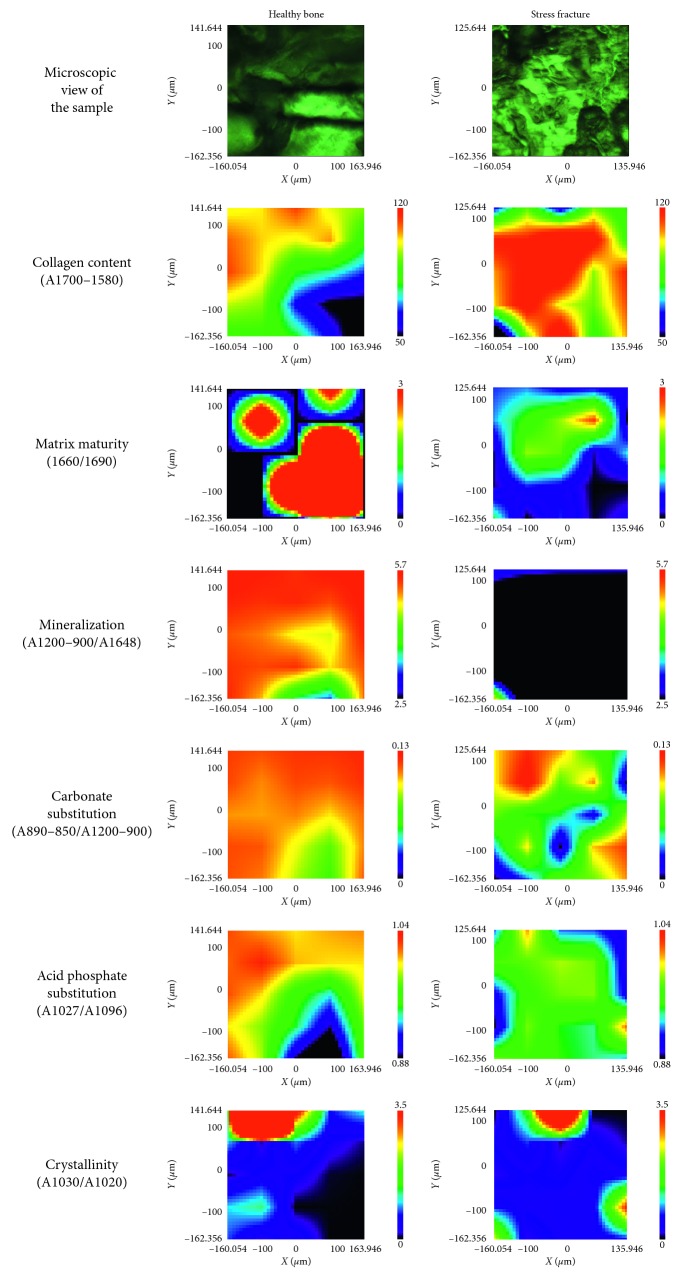
FTIR images in sections of healthy bone samples and bone samples with stress fracture. In all images, red color corresponds to the highest values and blue to the lowest values. Spatial distribution of the amount of collagen, as well as the matrix maturity, mineralization, carbonate substitution, acid phosphate substitution, and crystallinity ratios, was calculated from the FTIR spectra.

FTIRIs of the HB and bone with an SF are shown in Figure 3. Each image represents the integrated absorbance of a specific band or ratio of the IR spectra for each pixel of the MCT detector; red and blue colors represent strong and weak absorption, respectively, of the infrared beam. According to data mentioned above, the collagen content was higher in the bone with an SF; nevertheless, the matrix maturity, mineralization, carbonate substitution, and APS showed a higher intensity in the HB, but the CI exhibited higher intensity in the bone with an SF.

### 3.4. Histology

**Figure 4:**
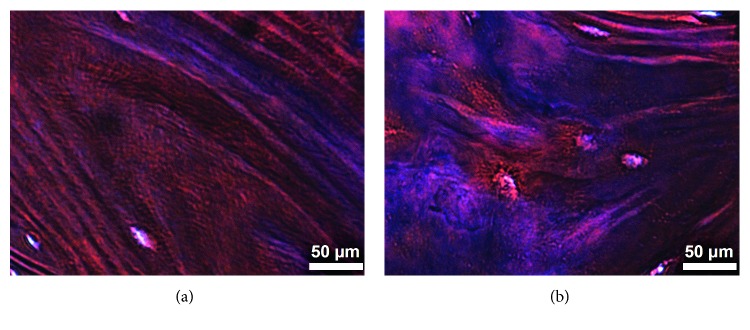
Longitudinal section of the metaphysis of the proximal femur (compact bone). The healthy bone showed normal collagen fiber staining, and the bone with stress fracture showed a greater amount of collagen fibers, which are disorganized. Masson's trichrome stain, 400x.

The histology examination employing Masson's trichrome stain in the femur metaphysis sections of the HB and SF groups is shown in Figure 4, evidencing in both groups histological characteristics of the compact bone, where some osteocytes are observed. Nevertheless, it is important to mention that, in the HB group, normal collagen fibers (stained in blue) of bone lamellae are evidenced (Figure 4(a)), and in the SF group, more collagen fibers are shown which are disorganized (Figure 4(b)).

## 4. Discussion

In this research, we investigated the biochemical composition (specifically collagen and mineral components) of bone samples with the diagnostic of the SF through FTIR spectroscopy and FTIRIs, analyzing their differences with respect to the healthy bone.

As previously mentioned, 11 bone samples from patients with the diagnosis of the SF were obtained, of which eight belonged to women, agreeing with the world literature, once it has been stated that women are at higher SF risk than men [3, 4], attributing this increased risk to the female athlete triad (menstrual irregularity, disordered eating, and osteopenia) [18].

In the same way, it is important to mention that ten patients with an SF were less than 30 years old; about this, Finestone and Milgrom [5] and Mosekilde [19] among other authors have declared that the age by itself is the principal determinant of bone strength, mass, and microarchitecture and also have stated that the maximum bone size and strength are reached at the age between 25 and 26 years. Moreover, men at the age of 20–30 years have a higher peak bone mass and strength than women.

On the contrary, as previously mentioned, these fractures have been well described in military recruits [1, 2], and in this research, ten of the 11 SF samples came from military recruits who were doing their basic military course. About this, Romani et al. have stated that SF risk may be highest during the third week after the onset of the new or increased activity, which is concordant with our results [2]. Therefore, most of the samples present well-established risks for SFs.

Nevertheless, Warden et al. among other authors have confirmed that SF susceptibility is directly related to skeletal properties, such as bone mass and size [1]. As previously mentioned, the mass refers to BMD, and the material properties involve the collagen and mineral components [1, 6, 7], the reason by which the biochemical profiles of these bone samples were analyzed through FTIR spectroscopy and FTIRIs.

Regarding the FTIR spectra analysis, the bone spectra of the HB and the bone with an SF exhibited intense bands in the collagen (1700–1200 cm^−1^) and phosphate (hydroxyapatite) (1200–900 cm^−1^) regions, being quite similar to those reported by Figueiredo et al., who characterized the molecular fingerprint of the HB through FTIR spectroscopy [20]. In the same way, they also agree with those reported by Paschalis et al. [7], highlighting that the HB group showed higher absorbance in the phosphate groups than the SF group, which could be related to the bone strength.

It is important to mention the band at 1745 cm^−1^ related to lipids was evidenced in this study; nevertheless, other authors have not exhibited this band in bone samples, maybe because most authors use ethanol to dehydrate the samples which removes fatty acids [21], and in this research, the samples were not fixed and were analyzed in the fresh state.

Moreover, During et al. have stated that the mineralized bone tissue itself contains lipids which play an essential role in bone physiology. It has been shown that fatty acids, cholesterol, phospholipids, and several endogenous metabolites act on bone cell survival and functions, as well as the bone mineralization process and critical signaling pathways [22]. However, Gamsjaeger et al. have stated that lipids have been reported as nucleators of collagen fiber mineralization, and a layer of lipids is present just behind the first mineral deposited [23]. So, lipids are very important in the bone mineralization process. In this sense, we can state that the SF group showed a lower lipid band absorption compared to the HB group because as expected, the SF has a poor bone mineralization.

As previously mentioned, the bone matrix is a two-phase system in which the mineral phase provides the stiffness and the collagen fibers provide the toughness. This is the reason by which the alterations in collagen fibers affect the mechanical properties of the bone and increase fracture susceptibility [24]. In this research, a greater collagen type I content was evidenced in the SF group, which was also concordant with Masson's trichrome stain, once in this group more collagen fibers were observed. About this, it is known that the SF is the result of microtraumas, circulatory compromise, and accelerated remodeling, provoking an increment in osteoclastic and osteoblastic activity. Moreover, in the normal bone remodeling process, osteoblasts generate a sufficient amount of bone, while osteoclasts' function is the bone absorption [25]. Nevertheless, in the SF, the remodeling process is accelerated, and there is not complete mineralization because the resorption begins before the bone remodeling process is finished. Therefore, a bone with an SF contains less inorganic (mineral) and more organic (collagen) components [26].

On the contrary, in the HB with age, there is an increase in matrix maturity, mineral content, carbonate substitution, and crystallinity [27, 28]. Snyder et al. have reported that a higher proportion of SFs occurred among younger subjects [18], which as previously discussed was also concordant with our results, once most of the study subjects who had an SF were younger than 30 years, the reason by which they have not reached their higher peak bone mass and strength. Furthermore, comparing the matrix maturity, mineralization, carbonate substitution, and crystallinity of SF samples with those of healthy patients around the same age, the SF samples presented changes in these ratios.

Regarding matrix maturity, it is known that bone toughness is supplied primarily by the organic matrix, and the task of stabilizing this polymeric network rests on a collection of covalent collagen cross-links [29, 30]. In this study, as expected, the cross-link ratio associated with matrix maturity was lower in the SF group, which is concordant with the results mentioned above about the relation of bone maturity and age. As mentioned above, insignificant shifts in the cross-link profile are associated with bone pathologies, the reason by which a lower matrix maturity ratio is correlated with bone fracture, low toughness, and strength.

Nevertheless, the collagen cross-link affects mainly the postyield properties of the bone, and the preyield strength is mainly dependent on the mineral phase, which was also assessed in this research through mineralization and carbonate substitution. With respect to the mineralization (represented by the mineral-to-matrix ratio), the SF samples showed a decrement in this ratio compared to the HB group, which is the result agreeing with that reported by Boskey and Pleshkocamacho who analyzed biopsies of patients with osteoporosis and healthy bones, showing a lower mineralization ratio in samples of osteoporosis (bones in fracture risk) [27]. In the same way, this also agrees with that reported by Gourion-Arsiquaud et al. who reported a substantial reduction in the mineral-to-matrix ratio in fractures of the femoral neck from women aged 65 to 91 years [31]. About this, it has been reported that bone strength depends on the matrix volume, microarchitecture, and also BMD. In the same way, Follet et al. have reported that, in a more mineralized bone, higher stiffness and compressive strength are shown [28]. Moreover, Warden et al. have stated that fractures resulting from bone insufficiency occur in bones that are mechanically compromised and generally present low BMD [1]. Besides, Boivin et al. stated that the mineralization degree of the bone is a determinant of its mechanical strength and hardness [32].

Nevertheless, it is important to mention that the term “mineralization” does not only involve the initial deposition of minerals in an organic matrix but also comprise their maturation until the upper mineral density in a given volume of the matrix is reached, including an increase in number, size, and perfection of crystals. Independently of bone mass and its distribution in space, the mineralization and the “quality” of the mineral play a crucial role in the elastic, plastic, and viscoelastic properties defining the mechanical behavior of bones [33]. In this regard, carbonate substitution was analyzed through carbonate-to-phosphate ratio, once it has been reported that carbonate substitution refers to the progressive transformation of immature surface-hydrated domains into a mature and more stable apatite lattice. About this, Boskey et al. have declared that low carbonate-to-phosphate ratios were attributed to a relative increase in the amount of minerals (phosphate) and aging [12]. However, in this research, we found a lower carbonate substitution ratio in the SF group compared to the HB group, which could be related to the low mineralization ratio. These results also agree with those reported by Isaksson et al., who analyzed renal osteodystrophic bone samples, reporting a lower carbonate-to-phosphate ratio compared to the HB, emphasizing that osteodystrophy results in a low bone strength [34]. Nevertheless, Boskey et al. have also stated that this ratio may not predict whether remodeling is increased or decreased, but instead, it indicates that the mineral content is abnormal, reflecting a typical area of older or microdamaged bone [12].

Moreover, during bone remodeling, after resorption, bone formation is a multistep process. Following the deposition, the new matrix begins to mineralize (the primary mineralization), and after full completion of the bone structural units, a secondary mineralization begins, in which a slow and gradual maturation of the mineral component, including an increase in the number and size of crystals and/or an increase of the perfection at the crystal level, is developed. The degree of mineralization is directly proportional to the hardness of the bone tissue; in this sense, poor mineralization decreases mechanical resistance, as occurring in osteomalacia [32, 35].

In this research, we used the APS ratio to evaluate the secondary mineralization [14]. Regarding this result, we can state that there is an alteration in the secondary mineralization in the SF group, once an increased amount of this component was observed in the SF group compared to the HB group. This poor secondary mineralization could explain the decrease in mechanical resistance, provoking stress fracture predisposition.

Concerning the CI (mineral crystal size and perfection), as expected, the crystallinity was higher in the SF group compared to the HB group. About this, it has been reported that crystallinity appears increased in the fractures [28], which is concordant with our results and also with results provided by Boskey and Pleshkocamacho who reported that, in biopsies of the bone from patients with osteoporosis, there are increases in crystallinity relative to the HB [27]. Similarly, our results agree with those reported by Gourion-Arsiquaud et al. who examined women with hip fracture and cadaveric bone as controls, reporting an increased crystallinity in the fracture group [31].

Regarding FTIRI analysis, all the results agree with the FTIR spectroscopic analysis results in the ATR mode, once the matrix maturity, mineralization, carbonate substitution, and APS were lower in the bone with an SF than in the HB. These results partially agree with those reported by Boskey et al. who investigated the association of FTIRI variables measured in iliac crest biopsies with fragility fractures, reporting a higher intensity in matrix maturity and crystallinity and lower intensity in mineralization and carbonate substitution in the bone fracture group [12]. Likewise, our results are partially concordant with those reported by Gourion-Arsiquaud et al. who analyzed through FTIRIs specific differences in spatially resolved bone composition that contribute to fracture risk, evaluating iliac crest biopsies of women with and without fractures, reporting that the parameters that were significantly associated with fracture were collagen maturity, mineralization, and crystallinity, which augmented with increased fracture risk. Nevertheless, in our results, the matrix maturity was higher in the HB; this is probably due to the fact that patients that Boskey et al. studied were between 49 and 79 years old and Gourion-Arsiquaud et al. analyzed women between 30 and 83 years old, and in this research, we considered patients from 18 to 40 years [12, 36]. Moreover, it is important to mention that Gourion-Arsiquaud et al. and Boskey et al. researches were in osteopenic patients, however, the stress fracture are not limited to an osteopenic condition. The SFs in young patients are more correlated with the bone immaturity.

Considering all those as mentioned earlier, we can state that there are quite differences between the FTIR spectra and FTIRIs of the SF and the reported FTIR spectra and FTIRIs of fragility fractures. These conditions have different physiopathologies, so the bone biochemical profile is different. Nonetheless, the two conditions showed lower mineralization and higher crystallinity.

Recent studies have indicated that the increased fracture risk in other metabolic bone diseases such as osteoporosis includes low bone mass, distorted bone structure, and altered composition of the bone tissue [12]. In the same way, poor nutrition and lifestyle habits may increase the risk of SFs [4].

Although it is not possible to prevent the SF, strategies of prevention may be considered, such as the screening tool based on recognition and modification of risk factors, as well as health promotion strategies including smoking cessation and the provision of advice appropriately [37].

## 5. Conclusions

In this study, FTIR spectroscopy and FTIRI analysis methods were used to show the biochemical differences between the bone with an SF and the HB. According to our findings, we conclude that these techniques probed that the SFs are related to the bone immaturity once the collagen content was increased and matrix maturity, mineralization, carbonate substitution, and acid phosphate substitution were decreased. Nevertheless, the development of some other noninvasive studies that allow the early identification of bone immaturity is necessary to establish prophylactic measures in patients prone to SFs.

## Figures and Tables

**Table 1:** Population characteristics in this study.

Patient	Sample type	Gender	Age	Military recruit
1	SF	M	24	^*∗*^
2	SF	F	27	^*∗*^
3	SF	F	20	^*∗*^
4	SF	F	27	^*∗*^
5	SF	M	22	^*∗*^
6	SF	F	19	^*∗*^
7	SF	F	19	^*∗*^
8	SF	F	32	
9	SF	M	26	^*∗*^
10	SF	F	21	^*∗*^
11	SF	F	21	^*∗*^
12	HB	M	36	^*∗*^
13	HB	M	24	^*∗*^
14	HB	F	30	
15	HB	M	18	
16	HB	F	21	^*∗*^

SF: stress fracture; HB: healthy bone; ^∗^corresponds to those studied patients that were military recruits.

## Data Availability

All the generated data and the analysis developed in this study are included in this article.
